# Safety of cholecystectomy performed by surgeons who prefer fundus first versus surgeons who prefer a standard laparoscopic approach

**DOI:** 10.1016/j.sopen.2024.04.004

**Published:** 2024-04-25

**Authors:** Åsa Edergren, Gabriel Sandblom, Mikael Franko, Thorhallur Agustsson, Yucel Cengiz, Gona Jaafar

**Affiliations:** a Department of Clinical Science and Education Södersjukhuset, Karolinska Institute & Department of Surgery, Södersjukhuset, Sjukhusbacken 10, 11883 Stockholm, Sweden; b Department of Clinical Science and Education Södersjukhuset, Karolinska Institute, Sjukhusbacken 10, 11883 Stockholm, Sweden; c Department of Surgical and Perioperative Sciences, Umeå University, 90185 Umeå, Sweden; d Department of Clinical Sciences, Intervention and Technology, Karolinska Institute & Department of Emergency Care, Karolinska University Hospital, Ana Futura, Alfred Nobels Allé 8, 141 52 Huddinge, Sweden

**Keywords:** Gallbladder surgery, Intraoperative complications, Cholecystectomy/adverse effects, Bile duct injury, Cohort studies

## Abstract

**Background:**

An alternative method to standard laparoscopic cholecystectomy (SLC) is the “fundus first” method (FFLC). Concerns have been raised that FFLC can lead to misinterpretation of important anatomical structures, thus causing complications of a more serious kind than SLC. Comparisons between the methods are complicated by the fact that FFLC is often used as a rescue procedure in complicated cases. To avoid confounding related to this we conducted a population-based study with comparisons on the surgeon level.

**Method:**

In GallRiks, the Swedish registry for Gallbladder surgery, we stratified all cholecystectomies performed 2006–2020 in three groups: surgeries carried out by surgeons that uses FFLC in <20 % of the cases (N = 150,119), in 20–79 % of the cases (N = 10,212) and in 80 % or more of the cases (N = 3176). We compared the groups with logistic regression, adjusting for sex, age, surgical experience, year of surgery and history of acute cholecystitis. All surgical complications (bleeding, gallbladder perforation, visceral perforation, infection, and bile duct injury) were included as outcome. A separate analysis was done with regards to operation time.

**Results:**

No difference in incidence of all surgical complications or bile duct injury were seen between groups. The rates of bleeding (OR 0.34 [0.14–0.86]) and gallbladder perforation (OR 0.61 [0.45–0.82]) were significantly lower in the “fundus first > 80% group” and the operative time was shorter (OR 0.76 [0.69–0.83]).

**Conclusion:**

In this study including >160,000 cholecystectomies, both methods was found to be equally safe.

**Key message:**

During laparoscopic cholecystectomy, the standard method of dissection and fundus first dissection are equally safe surgical techniques. Surgeons need to learn both methods to be able to use the one most appropriate for each individual case.

## Highlights

• Some surgeons fear that the fundus first technique in laparoscopic cholecystectomy has a higher risk for bile duct injury.
• We compared cases done by surgeons often using the standard technique with cases done by surgeons mostly using fundus first.
• No differences in the rate of all surgical complications or bile duct injury were found between groups.
• In this study, both methods appear equally safe.

## Introduction

Laparoscopic cholecystectomy (LC) is the method of choice for treatment of symptomatic cholelithiasis, and one of the most common surgical procedures in the European Union and the United States [1,2]. The standard method for LC (SLC) includes the creation of a critical view of safety, ligating the cystic duct and artery, and finally dissecting the gallbladder from the liver along the cystic plane [3,4]. An alternative to the standard method is fundus-first laparoscopic cholecystectomy (FFLC) (also called “fundus down”, “dome down”, or “top down”), where dissection starts from the fundus end of the gallbladder, similar to the technique often used in open surgery. This method was first described by Cooperman in 1990, who recommended it for cases with severe cholecystitis and adhesions around Calot's triangle [5]. Since then, several studies have investigated the use of the fundus-first technique in laparoscopic surgery, but they have often been small, or the level of evidence low. Strong evidence supporting one method over the other is currently lacking.

Bile duct injury (BDI) is the most feared complication of cholecystectomy since it has high lifelong morbidity and increased mortality [[6], [7], [8]]. The incidence of BDI after LC is 0.2–1.5 % [[9], [10], [11]]. Studies have found several risk factors for BDI after LC including surgical experience [12], hospital volume [13], obesity [14], emergency surgery [15], and impaired liver function [16]. However, the largest studies stress three main risk factors; acute cholecystitis, older age, and male sex [[12], [13], [14],^16^,^17^].

In 2020, an international conference on the prevention of BDI during cholecystectomy recommended SLC, despite the lack of evidence that SLC was safer than FFLC [18]. The same recommendation is found in the Tokyo Guidelines and the 2020 WSES guidelines for the detection and management of bile duct injury during cholecystectomy, both commenting that the quality of evidence for this recommendation is low [11,19]. Generally, SLC is recommended as standard procedure and FFLC as the alternative if a critical view of safety cannot be achieved [19,20]. There are studies reporting shorter operation times, fewer complications, and lower risk for conversion to open surgery when FFLC is used [[21], [22], [23]]. On the other hand, concerns have been raised that FFLC can make identification of important anatomical structures more difficult, leading to more serious complications (primarily bile duct injuries) than SLC, though these are very rare [24,25]. A report by Strasberg et al. from 2012 is widely quoted as showing the poor safety of FFLC, although this study only examined eight procedures, seven of which were converted to open surgery before any dissection commenced [24]. In contrast, a recent meta-analysis from 2022 based on 12 studies, including 4 randomised controlled trials (RCTs), showed a lower risk for BDI with FFLC (pooled risk ratio 0.21) and a lower risk for conversion to open surgery (pooled risk ratio 0.42). Operation time with FFLC was significantly shorter, and no significant difference in intraoperative gallbladder perforation rate was seen [26].

Cholecystectomy is usually planned as a SLC but may be converted to FFLC in the case of difficulty, such as in severe cholecystitis. Since the majority of FFLCs are performed in more complicated cases, simply comparing complication rates between SLCs and FFLCs is misleading. Moreover, most surgeons have less experience in FFLC, making surgical skill yet another confounder in such a comparison.

The primary aim of this study was to compare complication rates between SLC and FFLC where procedures were performed by surgeons with a preference for one or the other technique to circumvent the problems mentioned above. A secondary outcome analysis was operation time.

## Method

The study was conducted as a population-based study using data from GallRiks, the Swedish Register for Gallbladder Surgery and Endoscopic retrograde cholangiopancreatography (ERCP). GallRiks was started in 2005, and today covers >90 % of all cholecystectomies in Sweden.

Factors registered in GallRiks include sex, age, operation indication, surgical technique, American Society of Anesthesiologists (ASA)-classification, Body Mass Index (BMI), smoking, treatment with antibiotics, and intra- and postoperative complications [[27], [28], [29]].

To avoid confounding caused by intraoperatively switching from SLC to FFLC due to surgical difficulties, we designed this as a cohort study comparing procedures carried out by surgeons with a preference for FFLC with those by surgeons preferring SLC.

We began by identifying all cholecystectomies registered in GallRiks between 2006 and 2020 where the intention was to complete the procedure laparoscopically. We excluded patients younger than 18 years old. The procedures were then divided into three groups; one where the procedure was carried out by a surgeon performing FFLC in <20 % (N = 150,113), one where the surgeon performed FFLC in 20–79 % of cases (N = 10,211) and one where the surgeon performed FFLC in >80 % of cases (N = 3176). We then compared complication rates of the “less than 20% group” and the “over 80% group”.

We compared operation times and perioperative complications, adjusting for sex, age, presence of cholecystitis, year of surgery, and the total number of procedures performed by each surgeon.

### Statistical analysis

Analyses were performed using IBM SPSS Statistics version 29.0 (IBM Corp., Armonk NY) and R version 4.3.0.

All outcomes were analyzed using GEE models with variables above included as covariates and clustering components for hospital and surgeon. GEE allows for robust specification of correlation matrices and is more computationally efficient than standard linear and logistic regression for clustered data. For operation time, a log-transformation with a Gaussian link was used to account for the skewed distribution. A binomial link was used for the remaining binary outcomes. Age was divided into four categories: 18–39 years, 40–49 years, 50–64 years, and 65+ years. Year of surgery and total number of procedures were included as cubic splines with four degrees of freedom to account for the non-linear relationships. Results are presented as relative effects (RE) for operation time, and odds ratios (OR) for binary outcomes with 95 % confidence intervals and p-values.

### Variables analyzed

There are several variables for each complication registered in GallRiks, complications that could possibly be related to surgical technique, such as bleeding, as well as other complications such as postoperative thrombosis or cardiovascular complications. Complications are registered during or immediately following surgery, and at the thirty-day and six-month follow-ups. In this study we used the following variables: 1. *Intraoperatively confirmed complications*: defined as presence of either perforated intestine, bleeding requiring intervention, or BDI; 2. *Bleeding*: defined as bleeding requiring intervention such as transfusion or conversion to open surgery; 3. *Accidentally perforated gallbladder* or *perforated intestine*: included all perforations regardless of management. The same variables were registered at the thirty-day follow-up if the complication was discovered postoperatively. *Infection with abscess* was also included among the postoperative variables.

We analyzed each variable (bleeding, perioperative gallbladder perforation, perforation of intestine, BDI, and postoperative infection) separately, and also together as the variable “All surgical complications” including any of these complications. We also studied whether the year of surgery was associated with complication rate.

Operation times were also analyzed.

## Results

A total of 163,558 LC were registered in GallRiks between 2006 and 2020. Excluding 7 procedures performed on patients younger than 18 years and 51 lacking data on dissection, sex, or presence of cholecystitis, 163,500 cases were entered into the analysis (Table 1. Baseline characteristics). The procedures were performed by 2709 surgeons.

**Table 1:** Baseline characteristics.

	Cholecystectomies carried out by surgeons using FFLC in <20 % (N = 150,113)	Cholecystectomies carried out by surgeons using FFLC in 20 %–79 % (N = 10,211)	Cholecystectomies carried out by surgeons using FFLC in ≥80 % (N = 3176)	All cholecystectomies (N = 163,500)
Men	50,410 (33.6 %)	3520 (34.5 %)	1123 (35.4 %)	55,053 (33.7 %)
Women	99,703 (66.4 %)	6691 (65.5 %)	2053 (64.6 %)	108,447 (66.3 %)
Mean age, years (standard deviation)	50.7 (16.0)	50.5 (15.9)	49.5 (15.7)	50.7 (16.0)
Median age, years (IQR)	51 (38–63)	51 (38–63)	49 (37–61)	51 (38–63)
Cholecystitis	29,320 (19.5 %)	1967 (19.3 %)	597 (18.8 %)	31,884 (19.5 %)
Number of surgeons	2453	193	63	2709

Mean age, percentage of men, and patients with cholecystitis were similar in the three groups (Table 1).

The GEE models showed that sex, age, and presence of cholecystitis had a significant effect on the odds for “all surgical complications”, but there was no significant association with surgical technique (Table 2, all surgical complications). Similarly, there was no significant association between BDI and surgical technique (OR for FFLC 20–79 % 1.33 [0.88–2.01], or for FFLC >80 % 1.30 [0.91–1.84]).

**Table 2:** All surgical complications, multivariate analysis.

	Number of operations	Number of cases with any complication (%)	OR (95 % CI)	p-value
Total	163,500	4576 (2.8 %)	–	–
FFLC <20 %	150,113	4190 (2.8 %)	Ref	–
FFLC 20–79 %	10,211	291 (2.8 %)	1.07 (0.92–1.25)	0.39
FFLC ≥80 %	3176	95 (3.0 %)	1.06 (0.74–1.51)	0.75
Female	108,447	2714 (2.5 %)	Ref	–
Male	55,053	1862 (3.4 %)	1.16 (1.09–1.24)	<0.001
Age <40 years	45,422	844 (1.9 %)	Ref	
Age 40–49 years	32,577	683 (2.1 %)	1.10 (0.99–1.22)	0.089
Age 50–64 years	48,289	1389 (2.9 %)	1.48 (1.36–1.62)	<0.001
Age ≥65 years	37,212	1660 (4.5 %)	2.23 (2.04–2.43)	<0.001
No cholecystitis	131,616	3242 (2.5 %)	Ref	–
Cholecystitis	31,884	1334 (4.2 %)	1.51 (1.40–1.64)	<0.001

There was a significant association in the FFLC ≥80 % group between surgical technique and bleeding: OR 0.34 [0.14–0.86] (Table 3. Bleeding) and gallbladder perforation: OR 0.61 [0.45–0.82] (Table 4. Gallbladder perforation).

**Table 3:** Bleeding, multivariate analysis.

	Number of operations	Number of cases (%)	OR (95 % CI)	p-value
Total	163,500	1030 (0.63 %)	–	–
FFLC <20 %	150,113	944 (0.63 %)	Ref	–
FFLC 20–79 %	10,211	79 (0.77 %)	1.22 (0.81–1.84)	0.34
FFLC ≥80 %	3176	7 (0.22 %)	0.34 (0.14–0.84)	0.020
Female	108,447	533 (0.49 %)	Ref	
Male	55,053	497 (0.90 %)	1.56 (1.37–1.79)	<0.001
Age <40 years	45,422	146 (0.32 %)	Ref	–
Age 40–49 years	32,577	168 (0.52 %)	1.51 (1.21–1.88)	<0.001
Age 50–64 years	48,289	285 (0.59 %)	1.68 (1.37–2.06)	<0.001
Age ≥65 years	37,212	431 (1.16 %)	3.25 (2.65–3.98)	<0.001
No cholecystitis	131,616	722 (0.55 %)	Ref	–
Cholecystitis	31,884	308 (0.97 %)	1.56 (1.31–1.86)	<0.001

**Table 4:** Gallbladder perforation, multivariate analysis.

	Number of operations	Number of cases with gallbladder perforation (%)	OR (95 % CI)	p-value
Total	163,500	1030 (0.63 %)	–	–
FFLC <20 %	150,113	46,780 (31.2 %)	Ref	–
FFLC 20–79 %	10,211	2741 (26.8 %)	0.88 (0.73–1.08)	0.22
FFLC ≥80 %	3176	682 (21.5 %)	0.61 (0.46–0.80)	<0.001
Female	108,447	27,660 (25.5 %)	Ref	–
Male	55,053	22,543 (40.9 %)	1.78 (1.73–1.83)	<0.001
Age <40 years	45,422	10,270 (22.6 %)	Ref	–
Age 40–49 years	32,577	9155 (28.1 %)	1.25 (1.20–1.29)	<0.001
Age 50–64 years	48,289	16,152 (33.4 %)	1.55 (1.50–1.60)	<0.001
Age ≥65 years	37,212	14,626 (39.3 %)	1.80 (1.74–1.87)	<0.001
No cholecystitis	131,616	36,148 (27.5 %)	Ref	–
Cholecystitis	31,884	14,055 (44.1 %)	1.72 (1.63–1.80)	<0.001

Operation time was significantly shorter in the FFLC ≥80 % group: OR 0.76 [0.69–0.83]. Other factors that significantly affected operation time were sex, age, and presence of cholecystitis (Table 5. Operation time).

**Table 5:** Operation time, multivariate analysis.

	Number of operations	Median, minutes (IQR)	RE (95 % CI)	p-value
Total	163,500	90 (65–121)	–	–
FFLC <20 %	150,113	90 (65–121)	Ref	–
FFLC 20–79 %	10,211	89 (61–120)	1.03 (0.94–1.12)	0.51
FFLC ≥80 %	3176	90 (65–121)	0.76 (0.69–0.83)	<0.001
Female	108,447	85 (61–117)	Ref	–
Male	55,053	98 (70–133)	1.10 (1.10–1.11)	<0.001
Age <40 years	45,422	83 (60–113)	Ref	–
Age 40–49 years	32,577	87 (62–119)	1.03 (1.02–1.03)	<0.001
Age 50–64 years	48,289	90 (65–123)	1.06 (1.05–1.07)	<0.001
Age ≥65 years	37,212	98 (70–135)	1.12 (1.11–1.13)	<0.001
No cholecystitis	131,616	85 (61–115)	Ref	–
Cholecystitis	31,884	113 (83–150)	1.26 (1.23–1.28)	<0.001

## Discussion

In this population-based study including >160,000 cholecystectomies, no significant differences in the rates of “all surgical complications”, bile duct injury, intestinal perforation, or postoperative abscess were seen between surgeons preferring SLC and those preferring FFLC. The rates of bleeding (OR 0.34 [0.14–0.86]) and gallbladder perforation (OR 0.61 [0.45–0.82]) were significantly lower in the FFLC >80 % group. Moreover, we found significantly shorter operation times with FFLC (OR 0.76 [0.69–0.83]). This is in accordance with more recent studies [[21], [22], [23],^26^]. To our knowledge, this is the first study comparing SLC with FFLC at the surgeon level.

The strength of this study is that it includes a large number of cholecystectomies. A weakness is the small number of surgeons in the FFLC group (n = 63). This could be a confounder, as it cannot be excluded that these surgeons were more dedicated and skillful than those preferring SLC, even though we adjusted for experience.

It is notoriously difficult to compare surgical methods as there are so many factors to consider such as preference and level of skill of the surgeon, tradition, anatomical factors, and other patient factors. Randomised double-blinded studies are complicated by skewed figures and the need to deviate from protocol in the event of unforeseen complications. Observational studies may shed some light on the issue at hand from different points of view, eventually leading to appropriate conclusions. This study examines the safety of dissection technique in LC using a slightly different approach in that we attempt to remove the bias created by unfamiliarity of the surgeon with the technique in question.

## Conclusion

In this study comparing outcomes of two groups of surgeons preferring either the SLC or the FFLC technique, there were no differences in complication rates often feared by surgeon, both methods appear to be equally safe. Based on the results of this study, we recommend that surgeons familiarize themselves with both SLC and FFLC. This will enable them to choose the technique most appropriate for the case at hand. How can we expect to safely use FFLC as a bail-out strategy when the operation is complicated, if we never practice this method in a calm elective setting?

## Ethics approval

The study has been approved by the Swedish Ethics Review Authority (2021-04718).

## Funding sources

This study was funded with grants from 10.13039/501100004359Vetenskapsrådet/ the Swedish Research Council, grant number 2018-06926. The funding has been used for equipment and salary for the first author.

## CRediT authorship contribution statement

**Åsa Edergren:** Conceptualization, Formal analysis, Investigation, Project administration, Writing – original draft, Writing – review & editing. **Gabriel Sandblom:** Data curation, Formal analysis, Investigation, Supervision, Writing – review & editing. **Mikael Franko:** Data curation, Formal analysis, Methodology. **Thorhallur Agustsson:** Supervision, Writing – review & editing. **Yucel Cengiz:** Supervision, Writing – review & editing. **Gona Jaafar:** Conceptualization, Funding acquisition, Project administration, Resources, Supervision, Writing – review & editing.

## Declaration of competing interest

Dr. Sandblom is a board member for the Swedish Registry of Gallstone Surgery and Endoscopic Retrograde Cholangiopancreatography, GallRiks.

There are no other financial or personal relationships with other people or organizations that could inappropriately influence our work.
